# Impacts of commingling preconditioned and auction-derived beef calves on bovine respiratory disease related morbidity, mortality, and weight gain

**DOI:** 10.3389/fvets.2023.1137078

**Published:** 2023-03-17

**Authors:** Sanjaya Mijar, Frank van der Meer, Ed Pajor, Abigail Hodder, Julia Morgan Louden, Sean Thompson, Karin Orsel

**Affiliations:** ^1^Department of Production Animal Health, Faculty of Veterinary Medicine, University of Calgary, Calgary, AB, Canada; ^2^Department of Ecosystem and Public Health, Faculty of Veterinary Medicine, University of Calgary, Calgary, AB, Canada; ^3^Technology Access Centre for Livestock Production, Olds College Centre for Innovation, Olds College, Olds, AB, Canada

**Keywords:** fence-line weaning, preconditioning, auction-derived, mixing, health performance, average daily gain (ADG)

## Abstract

**Introduction:**

Stressors predisposing to bovine respiratory disease (BRD) upon arrival in the feedlot, include the ranch to feedlot transition and mixing cattle from multiple sources. Preconditioning (PC) reduces multiple stressors, but commingling PC and auction-derived (AD) calves in a feedlot may increase BRD risk. Our objective was to evaluate PC calf performance over the first 40 days in the feedlot and determine impacts of commingling with varying proportions of AD calves (25, 50, and 75%).

**Methods:**

Calves were either preconditioned at one ranch (*n* = 250) or mixed-source and bought from a local auction (*n* = 250). At arrival, calves were assigned into 1 of 5 pens: 100 PC, 75 PC, 50 PC, 25 PC, and 0 PC, reflecting the percentage of PC calves in a 100-head pen.

**Results:**

Over 40 days, morbidity in pen 100 PC was lower compared to 0 PC (24 vs. 50%, *P* < 0.001) and varied in commingled pens, being highest (63%) in 25 PC and least (21%) in 50 PC (*P* < 0.05). There were 3 AD deaths in 0 PC and 2 deaths in 25 PC. The AD calves in 0 PC were 3 times more likely to get BRD than PC calves in 100 PC; however, AD calves gained 0.49 kg/d more than PC calves (*P* < 0.0001). Ignoring pen placement, AD calves were 2.76 times more likely to get BRD but gained 0.27 kg/d more than PC calves (*P* < 0.0001). Commingling did not affect morbidity of PC (*P* = 0.5) or AD calves (*P* = 0.96), implying commingling did not affect health. Calves in 25 PC were 3.39 times more likely to get BRD than those in the 100 PC (*P* < 0.001). Furthermore, 25 PC calves gained the most (1.08 kg/d), followed by 50 PC (0.62 kg/d) and 75 PC (0.61 kg/d), compared to 100 PC (*P* < 0.05). Calf weight at arrival modified ADG (*P* < 0.05).

**Discussion:**

In conclusion, PC calves had lower morbidity over the first 40 days, irrespective of commingling. With larger variations in arrival weight, there was no advantage of PC for ADG in the first 40 days. The unknown weaning strategies and comparable arrival weights of AD calves may have contributed to greater ADG in AD calves.

## 1. Introduction

Bovine respiratory disease (BRD) is the predominant disease in the North American beef industry, causing substantial economic losses due to morbidity and mortality, treatments, reduced performance, and increased antimicrobial use (1, 2). Annual BRD incidence rate can be up to 44%, with estimated costs ranging from USD 13.90 to 151.18 per animal (3, 4). BRD is a multifactorial disease involving stress factors that predispose cattle to several viral and bacterial infections (5). These stressors pre-dominantly occur before and upon arrival at the feedlot, including an abrupt transition from ranch to feedlot, transportation, and mixing cattle from various sources. Pathogens most commonly implicated in BRD include *Mannheimia haemolytica, Pasteurella multocida, Histophilus somni, Mycoplasma bovis*, bovine herpesvirus 1 (BHV1), bovine parainfluenza 3 virus PI3V, bovine respiratory syncytial virus (BRSV), and bovine viral diarrhea virus (BVDV) (2, 6). In addition to deleterious effects of BRD on calf performance, health, and welfare, the common practice of metaphylactic antimicrobial use in feedlots is under scrutiny, due to implications for development of antimicrobial resistance in pathogens, food safety, and environmental sustainability (7).

Preconditioning (PC) to control BRD was proposed as early as 1967 (8) and constitutes management practices that reduce multiple stressors, including vaccination against bacterial and viral pathogens, optimized timing of dehorning, castration, best weaning strategy, and training calves to eat from a bunk and drink from a water source at least 45 d before transport to the feedlot (9, 10). Also, PC distributes stressors over time, rather than all at once, to improve calf performance in the feedlot.

It was reported that a 45-day post-weaning PC program can increase net profit $14 per head as compared to that were not preconditioned (11). Preconditioning reduced morbidity and mortality rates by 42.6 and 10.3%, respectively, compared to auction barn-derived calves in Kentucky (12). Despite positive impacts of PC, it is not widely implemented, or perhaps cow-calf producers just focus on vaccination and ignore other critical elements. For example, only ~42.5% of cow-calf operations provided calf health programs with preconditioning to buyers (13).

The Western Canadian Cow-calf Survey 2017 reported that only 22% of cow-calf producers preconditioned calves 30–60 days before selling to the feedlot operators (14). Additionally, the survey reported 70% of calves were sold *via* either live or electronic auction. Often, the history of management practices of calves sold at auction is unknown. Due to this limited availability of PC calves, feedlot operators often commingle PC and AD calves to fill pens based on weight, sex and frame comparison. However, commingling of calves from multiple sources favors the risk of BRD occurrence in the feedlot. For example, ranch sourced steers were less likely to be treated for BRD (morbidity 11%) than multi-sourced calves purchased through auction (morbidity 22%) in the feedlot (15). Commingling from multiple sources can increase the chance of exposure to infectious agents, suppresses immune responses due to stressors, and reduces calf health, performance and value compared to single-source calves (15). Similarly, BRD incidence was lower in pens commingling fewer vs. greater cattle sources (16). These studies, however, did not explore impacts of commingling and relative proportions of commingled PC and AD calves on health and performance soon after feedlot arrival.

Our objectives were to: (1) evaluate if PC calves perform better in terms of morbidity, mortality, and average daily gain than AD calves over the first 40 days (D40) in the feedlot; (2) evaluate impacts of optimized preconditioning on pen performance of morbidity, mortality, and average daily gain in the feedlot when commingled with various percentages (25, 50, and 75) of AD calves; and (3) evaluate impacts on PC and AD calves of commingling at various ratios.

## 2. Materials and methods

The protocol was approved by the University of Calgary Animal Care Committee (Animal Care Protocol AC20-0041). Cattle were cared for and used in accordance with the Canadian Council of Animal Care guidelines. The study design was described in detail (17) and is summarized briefly below.

### 2.1. Calves

Calves (*n* = 500) were acquired from two sources. Angus crossbred calves from a single ranch (WA Ranches of the University of Calgary) were designated as PC calves (*n* = 250), and the remainder were purchased from a local auction market (AD calves) in Olds, AB (*n* = 250).

#### 2.1.1. Preconditioned calves

The PC calves (*n* = 250) were selected based on birth date i.e., born from end of March to end of May 2020 (60-day interval) and ear-tagged within 24 h after birth. At ~60 days of age, they were given vaccines against common BRD pathogens and clostridial diseases: intranasal vaccine against BHV-1, PI3, and BRSV (Inforce 3-MLV, Zoetis, Parsipanny, NJ, USA), subcutaneous BVDV 1, 2 and *Mannheimia hemolytica* (Bovi-Shield Gold One Shot, Zoetis, Parsipanny, NJ, USA), 7-way clostridial vaccine (ULTRABAC 7/SOMUBAC, Zoetis, Parsipanny, NJ, USA), surgical castration, oral administration of meloxicam (3 ml/100 lbs), and a growth-promoting implant (SYNOVEX C, Zoetis, Parsipanny, NJ, USA).

At 3.5–5.5 months of age (late September 2020), calves were fence-line weaned for 5 days, with auditory and visual contact but no physical contact between calves and dams. Booster doses of BHV1, BRSV, PI3V (Inforce 3-MLV, Zoetis, Parsipanny, NJ, USA), BVDV1, 2 and *Mannheimia hemolytica* (Bovi-Shield Gold One Shot, Zoetis, Parsipanny, NJ, USA), and 7-way clostridial vaccine (ULTRABAC 7/SOMUBAC, Zoetis, Parsipanny, NJ, USA) were administered to the calves during the 5-day weaning period. Weaned calves were transported in a standard cattle liner to a pasture pen located 5 km away. They remained in the pasture-pen on feed and water for 45 days, and mid-November, the calves aged 5–7 months, were transported to the feedlot (17).

#### 2.1.2. Auction-derived calves

The AD calves (*n* = 250) were purchased from a local auction market after a presort from at least 24 consigners. Calves were visually appraised to match the PC calves, based on homogeneity of age, frame, breed, and weight. However, calf origin, vaccination, and timing and strategies of weaning were unknown. Calves were purchased on November 13^th^ and 14^th^, 2020 and transported to the feedlot, approximately 1 km from the auction market. One hundred of these calves spent 1 extra day at the auction, but were given access to feed, hay, and water.

### 2.2. Protocols at the feedlot

Preconditioned calves (PC) (*n* = 250) and AD calves (*n* = 250) were transported to the feedlot *via* standard cattle liners. The receiving protocol was in accordance with industry standards and included vaccination with Bovi-Shield Gold One Shot (Zoetis, Parsipanny, NJ, USA) and ULTRABAC 7/SOMUBAC (Zoetis, Parsipanny, NJ, USA), IVOMEC (Boehringer Ingelheim, Ingelheim, Germany), and a growth-promoting implant SYNOVEX C (Zoetis, Parsipanny, NJ, USA).

No antimicrobial was provided on arrival. The calves were screened for any clinical signs of BRD, which included a rectal temperature of 40°C or higher, cough, nasal discharge and/or difficulty breathing, and treated with antibiotics if diagnosed with BRD. A treatment protocol for the calves with clinical signs of BRD was followed. First pulled calves were treated with Florfenicol and Flunixin Meglumine (Resflor Gold, Merck, Madison, NJ, USA). If clinical signs persisted for 72 h, calves were given enrofloxacin at a dosage rate of 7.5–12.5 mg/kg BW (Baytril 100, Bayer HealthCare LLC, Shawnee Mission, Kansas, USA). If clinical signs persisted for another 72 h after that treatment, calves were treated with Trimethoprim-Sulfadoxine at a dosage rate of 1,000 mg/15 kg BW (Trimidox, Vetoquinol N.-A. Inc., Lavaltrie, Quebec, Canada). Calves that died during the 40-day experimental period were necropsied. Individual body weight was recorded on the day of arrival (weight in; D0) in the feedlot and on D40 (weight out). The average daily gain (ADG) of calves over 40 days was based on the weight in and weight out [ADG = Weight out-Weight in/Days on feed (DOF)]. ADG was expressed as kilogram/day (kg/d). Calves that died were removed from the data in the calculation of ADG on a pen level. Morbidity and mortality were expressed as percentages.

### 2.3. Pen allocation at the feedlot

**Figure 1:**
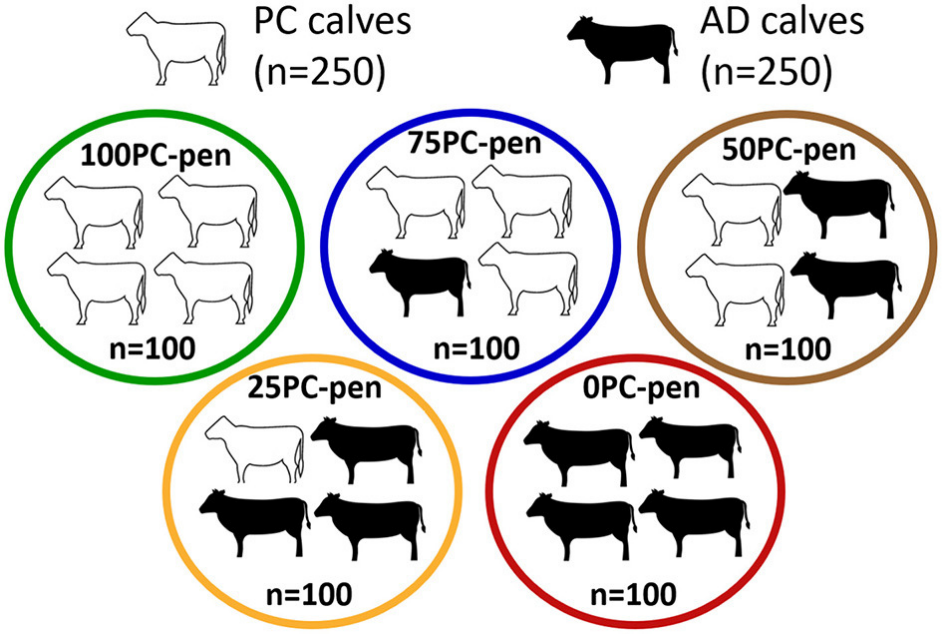
Schematic representation of calf allocations in each pen. PC, preconditioned calves; AD, auction-derived calves.

On November 13^th^, 150 PC calves arrived at the feedlot, of which the first 100 that entered the chute, irrespective of age or weight, were placed in pen 100 PCand 50 were placed in 50 PC. Additionally, 150 AD calves arrived, of which 100 were placed in 0 PC and 50 were placed in 50 PC. On November 14th, 100 PC calves arrived, of which 75 were placed in 75 PC and 25 were placed in 25 PC. Of the 100 AD calves that arrived the next day, 75 were placed in 25 PC and the remaining 25 were placed in 75 PC (Figure 1). The details of sources of calves filling the pen is provided in Table 1.

**Table 1:** Details of filling of pen with source of calves received at the feedlot.

**Pen**	**Source**	**Date received**	**No. of calves placed**		**Mean ±SD arrival BW (kg)**
			**PC**	**AD**	
100 PC	WA Ranches	November 13, 2020	100		288 ± 27^a^
50 PC	WA ranches/auction market	November 13, 2020	50	50	268 ± 25^b^
0 PC	Auction market	November 13, 2020		100	277 ± 16^b^
75 PC	WA ranches/auction market	November 14, 2020	75	25	281 ± 29^ab^
25 PC	WA ranches/auction market	November 14, 2020	25	75	282 ± 24^ab^

^a, b^Within a column, means without a common superscript differed (*P* < 0.05). PC, preconditioned calves; AD, auction-derived calves.

### 2.4. Statistical analyses

Data was analyzed using STATA (Ver. 16.1; StataCorp LLC, College Station, TX, US). Normality was tested by Shapiro-Wilk W tests. Two-sample Student's *t*-tests were used to detect differences between two normally distributed groups, whereas for non-normally distributed data, two-sample Wilcoxon rank-sum (Mann-Whitney) tests were used. For regression modeling, ADG, morbidity, and mortality were outcomes of interest and pen, source (PC vs. AD), and arrival weight were included as explanatory variables. In univariate analyses, *P* > 0.2 was a cut-off for explanatory variables to be considered in the full model. The ADG was analyzed with multivariable linear regression, whereas morbidity and mortality were analyzed with multiple logistic regression. The referent group was chosen for categorical variables based on biological reasoning. Variables identified as confounders were kept in the model if they resulted in a >20% change in the estimate when removed (backward elimination).

## 3. Results

**Figure 2:**
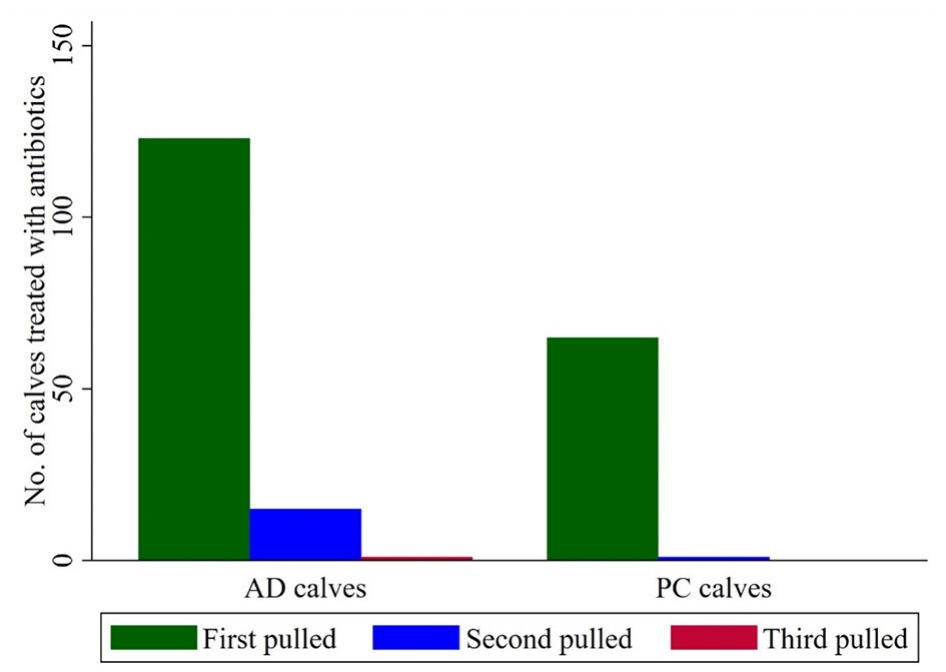
Number of calves treated with antibiotics over D40 at the feedlot. First pulled-calves those were sick for the first time, pulled and treated with an antibiotic. Second pulled-calves whose disease signs persisted for >72 h after the first antibiotic treatment. Third pulled-calves whose disease signs persisted for >72 h after the second antibiotic treatment.

**Figure 3:**
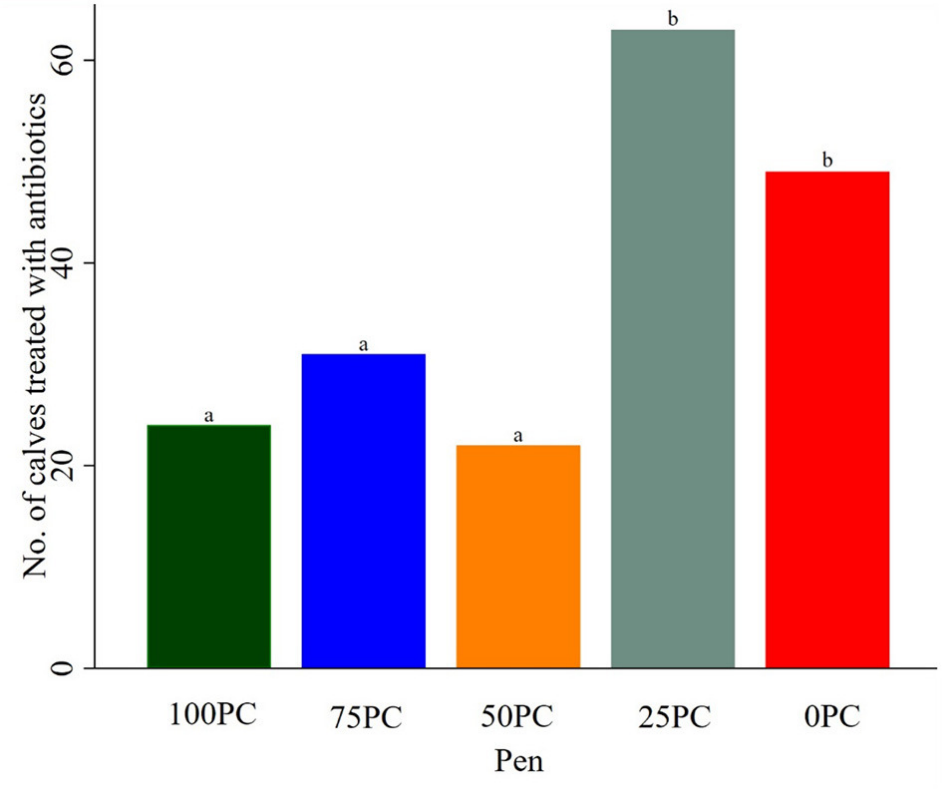
Number of calves (*n* = 190) treated in pens irrespective of source. PC reflects number of preconditioned calves among a group of 100. ^ab^Counts without a common superscript differed (*P* < 0.05).

Of the 500 calves, 190 (38%) were diagnosed with BRD over D40, of which 66 were PC and 124 were AD. Of the 217 total treatments for BRD, 28 calves were pulled twice (27 AD and one PC), and one was pulled three times (AD calf; Figure 2). Two calves were considered chronically ill and were moved to a dedicated sick pen (one PC calf from 75 PC-pen, one AD calf from the 25 PC-pen) and removed from the study results. Among calves in commingled pens, 63 in 25 PC required antibiotic treatments followed by 31 in 75 PC and only 22 in 50 PC. More calves in 0 PC than 100 PC required antibiotic treatments (50 vs. 24, *P* < 0.001), whereas more calves in 25 PC than 100 PC were treated with antibiotics (*P* < 0.001; Figure 3). At a pen level, morbidity was highest in 25 PC and least in 50 PC (63 vs. 21%, respectively). Five AD calves died or were humanely euthanized within D40 of the trial, three in 0 PC and two in 25 PC. Mean ADG was highest (1.43 kg/d) in 0 PC and the least (0.91 kg/d) in 75 PC.

We present three comparisons, starting with a PC and AD comparison irrespective of pen allocation, pen comparisons to 100 PC, and finally PC and AD comparisons including pen allocation, reflecting commingling ratios.

### 3.1. Comparison of morbidity and ADG of preconditioned or auction-derived calves irrespective of pen placement

When comparing 100 PC (PC only) and 0 PC (AD only) pens, AD calves were 3.00 times more likely to develop BRD than PC calves (*P* < 0.001). In contrast, AD calves gained an average of 0.49 kg/d more than PC calves (*P* < 0.0001) during 40 d at the feedlot. Upon comparing between all PC (*n* = 250) and all AD calves (*n* = 250), irrespective of their placement in the study, AD calves were 2.76 times more likely to get BRD than PC calves (*P* < 0.0001). In addition, AD calves gained an average of 0.27 kg/d more than PC calves (*P* < 0.0001).

### 3.2. Comparing a pen of preconditioned calves (100 PC) with pens commingled at different ratios for morbidity and ADG

Overall, adjusted for confounding effects of source, calves in 25 PC were 3.39 times more likely to get BRD than 100 PC calves (*P* < 0.001). Furthermore, calves in 100 AD were more likely to get BRD (OR = 1.70, *P* > 0.05) than those in either 50 PC (OR = 0.64, *P* > 0.05) or 75 PC (OR = 1.15, *P* > 0.05) as compared to calves in 100 PC-pen; however, these adjusted estimates were not significant (Table 2).

**Table 2:** Final multivariate logistic regression model for morbidity related to BRD at pen level by source.

**Predictor**	**Odds Ratio**	**95% CI**	***P*-value**
Intercept	0.59	0.29 ± 1.2	0.15
**Pen**			
100 PC	Referent		
0 PC	1.70	0.75 ± 3.81	0.2
75 PC	1.15	0.60 ± 2.20	0.69
50 PC	0.64	0.31 ± 1.33	0.24
25 PC	3.39	1.63 ± 7.04	< 0.001
**Source**			
AD	Referent		
PC	0.54	0.32 ± 0.93	< 0.05

PC, preconditioned calves; AD, auction-derived calves.

**Figure 4:**
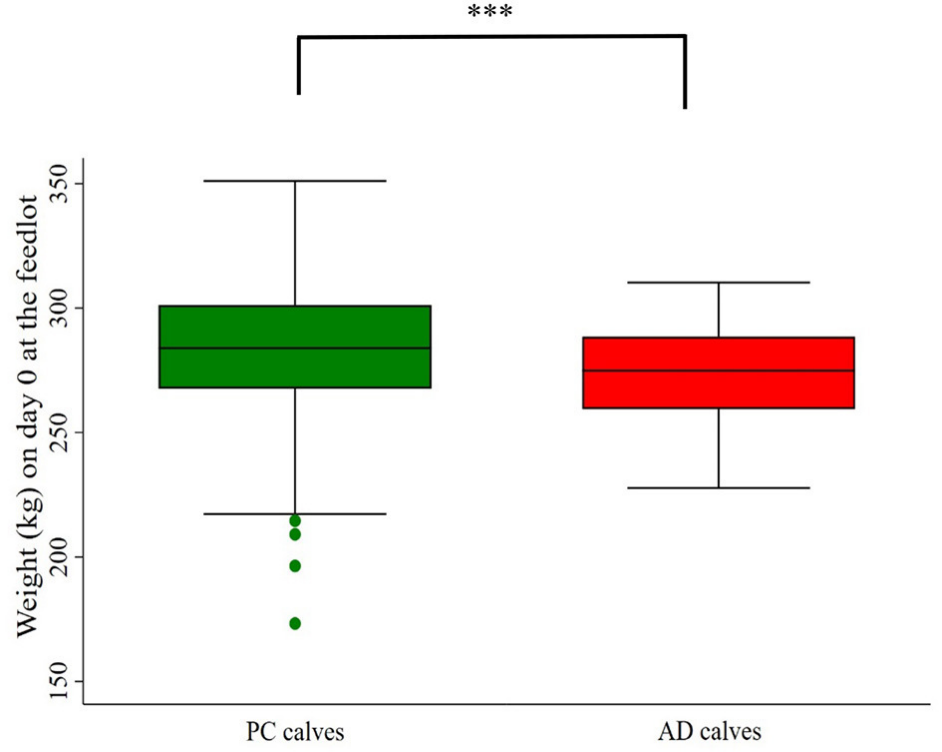
Body weight of calves on day 0 at the feedlot. PC, preconditioned calves. AD, auction-derived calves; ***Difference (*P* < 0.001).

**Figure 5:**
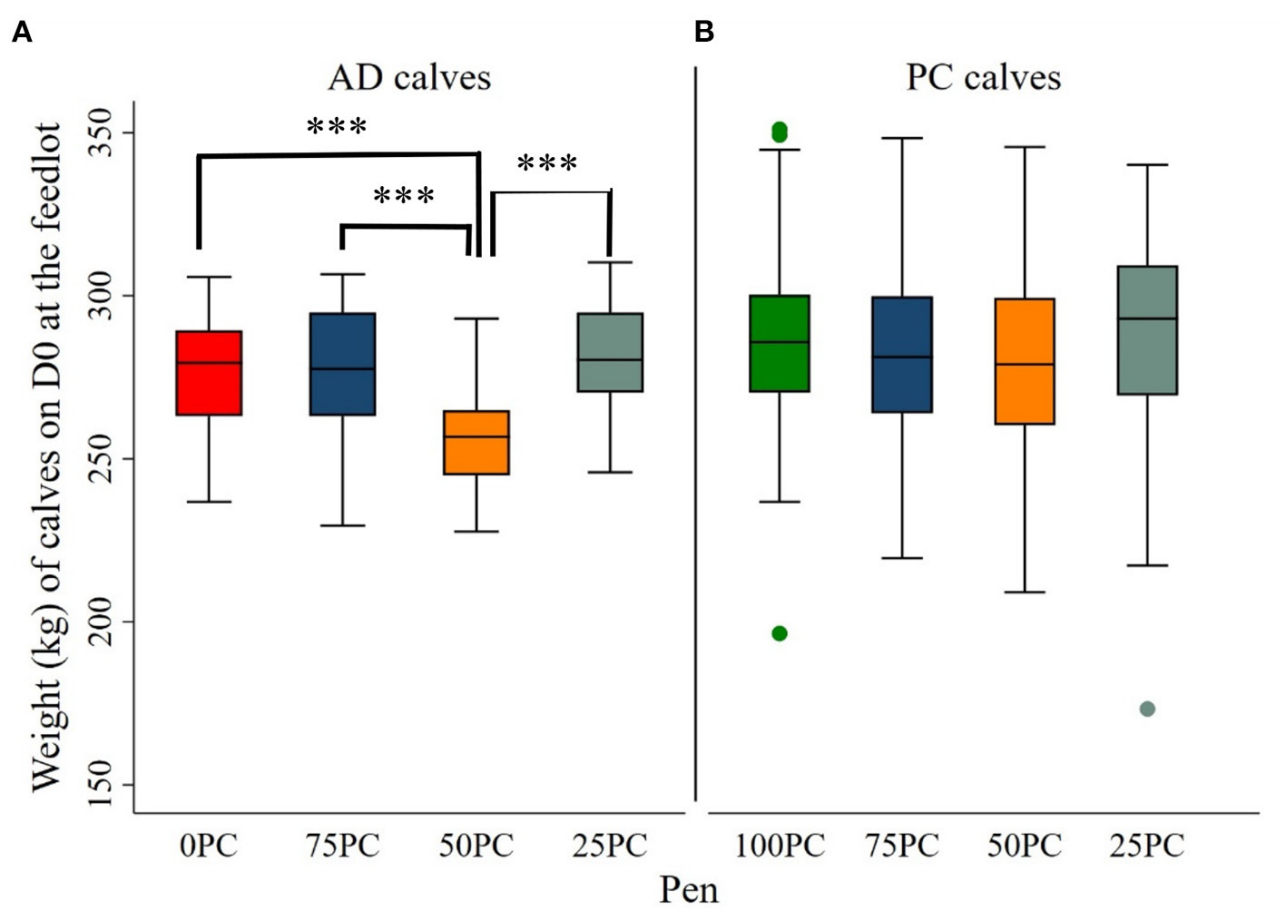
Body weight of calves on D0 at the feedlot. **(A)** Represents auction-derived (AD) calves in each pen. **(B)** Represents preconditioned (PC) calves in each pen. ^ab^Counts without a common superscript differed (*P* < 0.0001).

Source (PC vs. AD) had a confounding effect on ADG within a pen. Calves in 75 PC gained 0.60 kg/d more than those in 100 PC (*P* < 0.05). Similarly, calves in 50 PC gained 0.60 kg/d more than 100 PC (*P* < 0.01), calves in 25 PC gained 1.04 kg/d more than 100 PC (*P* < 0.05), and calves in 0 PC-pen gained 0.11 kg/d more than those in 100 PC-pen (*P* > 0.05). There was a significant effect modification between pen and arrival weight that affected ADG of calves among pens (Table 3). The details of feedlot arrival weight of calves on different sources and among pens are provided in Figures 4, 5.

**Table 3:** Final multivariate linear regression model for average daily gain at pen level by source and arrival weight.

**Predictor**	**Coefficient**	**95% CI**	***P*-value**
Intercept	0.65	0.15 ± 1.15	0.01
**Pen**			
100 PC	Referent		
0 PC	0.11	−0.18 ± 0.39	0.46
75 PC	0.60	0.04 ± 1.17	< 0.05
50 PC	0.60	0.30 ± 0.91	< 0.0001
25 PC	1.04	0.20 ± 1.88	< 0.05
**Source**			
AD	Referent		
PC	−0.08	−0.17 ± 0.01	0.07
Arrival weight	0.003	0.0001 ± 0.007	< 0.05
Pen × arrival weight	−0.001	−0.002 ±−0.0001	< 0.05

PC, preconditioned calves; AD, auction derived calves.

### 3.3. Impact of commingling in terms of morbidity and ADG

#### 3.3.1. Comparison of PC calves in 100 PC with PC calves in commingled pens

While comparing if PC calves in the single source pen performed better than PC calves in all the commingled pens combined as a means of evaluating impacts of commingling, PC calves in 100 PC were less likely to get BRD than any PC calves in commingled pens; however, this association was not significant (OR = 0.83, *P* = 0.5). However, PC calves in 100 PC gained 0.07 kg/d less than PC calves in combined commingled pens (*P* < 0.05).

When comparing PC calves in 100 PC vs. PC calves in pens with various commingling ratios (75 PC, 50 PC, and 25 PC), there were no significant differences in morbidity between the specific pens.

Upon comparison of ADG between PC calves in 100 PC-pen and PC calves in all three commingled pens, PC calves in 100 PC gained 0.24 kg/d less than PC calves in 50 PC (*P* < 0.0001) and 0.12 kg/d less than PC calves in 25 PC (*P* < 0.05). However, PC calves in 100 PC gained 0.04 kg/d more than PC calves in 75 PC (*P* = 0.17).

#### 3.3.2. Comparison of AD calves in 0 PC with AD calves in commingled pens

There was no significant difference in morbidity between AD calves in 0 PC vs. those in all commingled pens combined (*P* = 0.96). However, AD calves in 0 PC gained 0.29 kg/d more than AD calves in any commingled pen (< 0.0001).

When comparing if AD calves in 0 PC had higher morbidity than AD calves in commingled pens, AD calves in 0 PC had higher morbidity than AD calves in 75 PC or 50 PC (OR = 2.1, *P* < 0.05 and OR = 2.66, *P* < 0.01, respectively). However, AD calves in 0 PC had higher morbidity than AD calves in 25 PC (OR = 0.37, *P* < 0.01).

When comparing ADG between AD calves in 0 PC vs. commingled pens, AD calves in 0 PC gained 0.48 kg/d more than AD calves in 75 PC (*P* < 0.0001) and 0.36 kg/d more than AD calves in 25 PC (*P* < 0.0001). However, ADG in AD calves in 0 PC vs. 50 PC did not differ (*P* = 0.128).

## 4. Discussion

In this study, irrespective of ratio of commingling, PC calves had lower morbidity than AD calves over 40 days at the feedlot. Furthermore, pen placement did not significantly affect morbidity of PC calves. Therefore, we inferred that commingling did not affect BRD morbidity.

When comparing single-source PC calves to pens with calves of both sources at varying ratios, only the pen with 25% PC calves had significantly higher BRD morbidity. Furthermore, if we only compared PC calves across pens, there was no significant difference in BRD morbidity. Despite industry reluctance to purchase PC calves if pens cannot be filled with same-source calves, PC calves outperformed regardless of mixing with AD calves.

The AD calves had higher morbidity and therefore required more antibiotic treatments than PC calves. Additionally, most second- and third-time pulls for antibiotic treatments were AD calves. Similarly, ranch-sourced calves were less likely to be treated than market-purchased and commingled calves (15). The current study provided insights into how AD calves needed antibiotics more frequently to regain health compared to PC calves. Additionally, the frequency of antibiotic use was highest in a pen with a higher proportion of AD calves commingled. Alternatively, PC reduced antimicrobial use, despite commingling with calves from other sources. Although we did not characterize antimicrobial resistance, repeated antibiotic treatments likely promote bacterial antibiotic resistance.

Although overall mortality in our study was low, only AD calves died. Despite the limited power to study impacts of commingling on mortality, AD calves had a greater tendency for mortality due to BRD than PC calves and morbidity could be more in commingled vs. non-commingled pens. Similarly, in a previous study (12), mortality was greater in AD vs. PC calves.

In our study, AD calves had a higher ADG than PC calves, independent of commingling. In a previous study, there was no difference in ADG between ranch-sourced, auction market, and commingled calves at 42 d after being received at the feedlot (15). However, in an ideal situation, PC calves have a higher ADG than AD calves due to reduced stress. In our study, although PC calves had a higher weight on D0 at the feedlot than AD calves, the wider range of arrival weight for PC calves may have contributed to the higher ADG in AD calves over D40 at the feedlot. In contrast, weight of AD calves on D0 had a narrower range and were more uniform than PC calves. These negative results may have been related to sorting our PC calves at birth, which caused a wide variation in arrival weight when they arrived at the feedlot. However, AD calves that were purchased from a local auction market were more similar in BWT as they were sorted based on body size/frame. Additionally, AD already experienced being placed in a new environment, so at least a second exposure to a new environment might have placed them in a more favorable position, as PC calves were completely naïve and can be identified as neophobic, where neophobic is refered to as the fearful reaction to novel stimuli or situtions (18).

Additionally, another possible reason for higher ADG in AD calves than PC calves could be the use of antimicrobials in diseased calves at the feedlot. Since we did not use antimicrobials on arrival at the feedlot as prophylaxis, morbidity was higher in AD and hence required antibiotic treatment. All treated calves were included in the study for ADG; however separate analysis of only non-treated animals did not yield other outcomes (results not shown). Several studies have demonstrated that the use of antimicrobials in high-risk of BRD incidence in calves perform better by achieving desired feed and water intakes, which could have contributed to the higher gains identified in this study (10, 19, 20).

In our study, there was minimal shrinkage expected in AD calves purchased from a local auction market, as the calves were provided sufficient hay and water in auction pens before being transported to the feedlot. In addition, purchased AD calves were of British breeds and comparable in performance to WA Ranch-sourced PC calves. Another potential reason for higher ADG in AD calves could be due to inclusion of PC calves from a single ranch with their genetic potential. Moreover, many factors of AD calves such as weaning, vaccination status and nutrition before marketing of AD were unknown. It is impossible to assess the influence of these factors on the outcome. Replication of this study design could increase power and external validity of the findings. However, our study emphasized that BRD may be prevented by PC and minimized antimicrobial use in the feedlot. There is also a need for further exploration on ADG differences in calves of different sources in the feedlot, based on extensive commingling and PC protocol.

## 5. Conclusions

In general, calves that were preconditioned, i.e., fence-line weaned, vaccinated, castrated, and dehorned, bunk feed trained at least 45 d before being transported to the feedlot were less likely to get BRD as compared to calves purchased at an auction market. Irrespective of ratios of commingling with AD calves, PC calves consistently had lower BRD morbidity. Antimicrobial use was higher in AD calves than PC calves and in pens with a higher proportion of AD calves commingled. Therefore, PC reduced BRD and antimicrobial use in the feedlot, in commingling or non-commingling settings, as PC calves consistently performed better than AD calves, irrespective of commingling. The lower ADG in PC calves compared to AD calves was associated with arrival weights but could also be due to unknown factors of AD calves, including weaning strategy, vaccination, and nutrition.

## Data availability statement

The datasets presented in this article are not readily available because parts of the dataset will be used for another publication and cant be shared till publication. Requests to access the datasets should be directed to karin.orsel@ucalgary.ca.

## Ethics statement

The animal study was reviewed and approved by University of Calgary Animal Care Committee (Animal Care Protocol AC20-0041).

## Author contributions

KO, EP, and SM conceptualized the study. AH, JL, FvdM, and ST contributed to study design and sample collection. All authors listed have made substantial contributions in developing the manuscript.
